# A preoperative parathyroid scan is important for the total removal of the transplanted parathyroid tissue in recurrent secondary hyperthyroidism: A case report and literature review

**DOI:** 10.1097/MD.0000000000032453

**Published:** 2022-12-23

**Authors:** Xin-Ling Guo, Wen-Yao Yin

**Affiliations:** a School of Medicine, Tzu Chi University, Hualien City, Taiwan; b Department of General Surgery, Buddhist Tzu Chi Medical Foundation Dalin Tzu Chi Hospital, Chia Yi, Taiwan.

**Keywords:** autotransplantation, parathyroid scan, recurrent secondary hyperparathyroidism

## Abstract

Secondary hyperparathyroidism was one of mineral and bone disorders owing to chronic kidney disease. Patients who suffer from secondary hyperparathyroidism would receive medical treatment or parathyroidectomy with or without autotransplantation (AT). However, some patients receiving parathyroidectomy with AT have recurrent hyperparathyroidism, which impacts their lives. Patients with recurrent hyperparathyroidism may present persistent hypercalcemia and hyperphosphatemia, which would cause cardiovascular disease, like atherosclerosis.

**Patient concerns:**

A 63-year-old female of Asian descent with chronic kidney disease who suffered from recurrent hyperparathyroidism for twice. The patient underwent parathyroidectomy with AT in the left thigh when secondary hyperparathyroidism happened. After 3 months, recurrent hyperparathyroidism happened.

**Diagnosis:**

The patient was diagnosed with recurrent hyperparathyroidism due to chronic kidney disease with hyperparathyroidism status post parathyroidectomy with AT in the left thigh. Our patient also suffered from mineral and bone disorder.

**Intervention:**

Two parathyroid adenoma in the left thigh were found. However, one of them was too small to found in the operation. Therefore, autograftectomy of the large one was performed. However, hyperparathyroidism happened again. This time, the autograftectomy was performed under dual phase Tc-99m MIBI (99m Tc-methoxy isobutyl isonitrile) parathyroid scintigraphy and it succeeded.

**Outcomes:**

After secondary autograftectomy, the value of intact parathyroid hormone was surveyed immediately and dropped by two-third followed by gradual reduction in the following weeks. The calcemia and phosphatemia were back to normal gradually.

**Lessons:**

In our case, importance of scintigraphy in the parathyroidectomy was confirmed.

## 1. Introduction

Secondary hyperparathyroidism is one of the complications of chronic kidney disease (G3a-G5D). About 20 to 80% of all patients with chronic kidney disease suffer from secondary hyperparathyroidism. Mineral and bone disorder which is caused by chronic kidney disease would lead to hypocalcemia, hyperphosphatemia, calcitriol deficiency, elevated fibroblast growth factor-23. Due to dysregulation mineral metabolism, parathyroid gland hyperplasia occurs and over stimulation of parathyroid hormone (PTH). Severe hyperparathyroidism can lead to renal osteodystrophy, pruritus, muscular pain, tissue calcifications, osteitis fibrosa, and fractures. Hypercalcemia and hyperphosphatemia caused by secondary hyperparathyroidism would lead to vascular and myocardial calcification, which would be associated with high risk of cardiovascular risk.

According to The Kidney Disease: Improving Global Outcomes (KDIGO) 2017 Clinical Practice Guideline Update for the Diagnosis, Evaluation, Prevention, and Treatment of chronic kidney disease–mineral and bone disorder (CKD-MBD), there are some recommendations for treatment of abnormal PTH (PTH) levels in CKD-MBD. In patients with CKD (chronic kidney disease) G5D requiring PTH-lowering therapy, calcimimetics, calcitriol, or vitamin D analogs, or a combination of calcimimetics with calcitriol or vitamin D analogs. However, patient with G3a-G5D with severe hyperparathyroidism who fail to respond to medical or pharmacological therapy, they suggest parathyroidectomy. However, recurrent hyperparathyroidism happened after some patients received parathyroidectomy, which would impact their lives. Therefore, there are many investigations and serum tests preventing recurrent or persistent.

## 2. Case report

A 63-year-old housewife of Asian ethnicity with underlying disease of hypertension and end stage renal disease. She has been in the stage V CKD without dialysis for several months. She also had secondary hyperparathyroidism. Initially, she was only under symptomatic medical treatment. However, high intact PTH (iPTH) persisted, and did not respond to medical treatment. Therefore, she received parathyroidectomy plus left thigh transplantation at other teaching hospital on 20200123. After the surgery, she was diagnosed as hungry bone syndrome with severe hypocalcemia (1.51 mmol/L, normal 2.15–2.58 mmol/L). Due to the severe condition, she received calcium supplement. However, she suffered from general weakness and multiple joints pain and sought medical help at emergency room on 20200131 1 week after operation. Hypercalcemia was noted and bone syndrome were presented. In physical examination, no definite abnormal mass was palpable nor seen moving with swallowing action. In contrast, an oblong shaped nontender measuring about 2 cm length firm to hard mass consistency was palpable on anteromedial aspect of the left thigh under her previous transplanted site. Recurrence of secondary hyperparathyroidism was suspected.

Laboratory investigations showed iPTH of 592.98 (pg/ml). Considering recurrent hyperparathyroidism, she received medical treatment of Denosumab, vitamin D, CaCO3, Cinacalcet. However, persistent hypercalcemia, hyperphosphatemia, and hyperparathyroidism (iPTH:2584.07pg/ml) was noted although under medical treatment. Bone syndrome exacerbated, like bilateral rotator cuffs tear. (see supplement 1, Supplemental Digital Content, http://links.lww.com/MD/I221, which presented physiological change in 1 year after parathyroidectomy with autotransplant).

**Figure 1.**
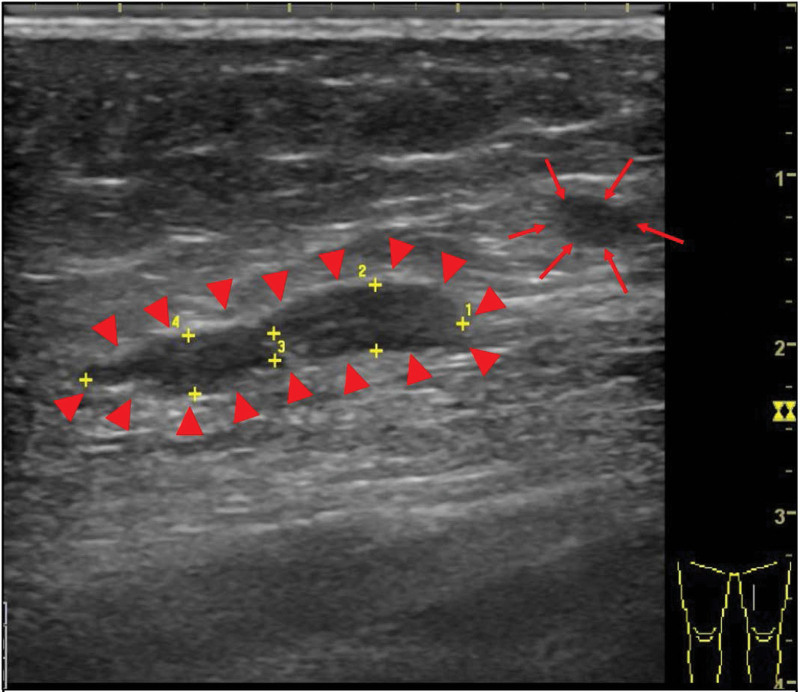
The preoperative sonography before first autograftectomy showed 2 hypoechoic masses. The larger one (1.7 cm × 0.4 cm, arrow head) and the smaller one (0.6 × 0.4 cm, arrow) at adjacent area.

**Figure 2.**
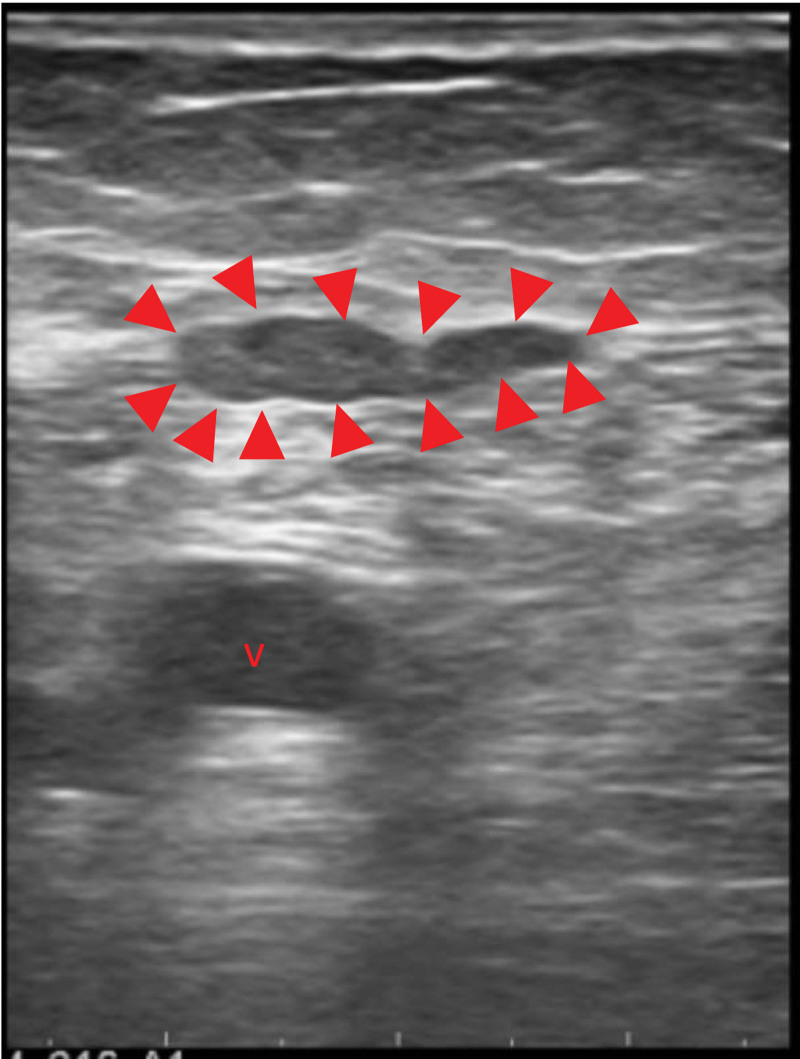
The intraoperative sonography during the first autograftectomy showed only the larger parathyroid mass (1.7 7cm × 0.37cm, arrow head) and was removed, but the smaller one was not definitely identified under sonography during operation and was left behind with the idea that even if the small remnant tissue not being removed might not be harmful afterwards. (v: vessel).

**Figure 3.**
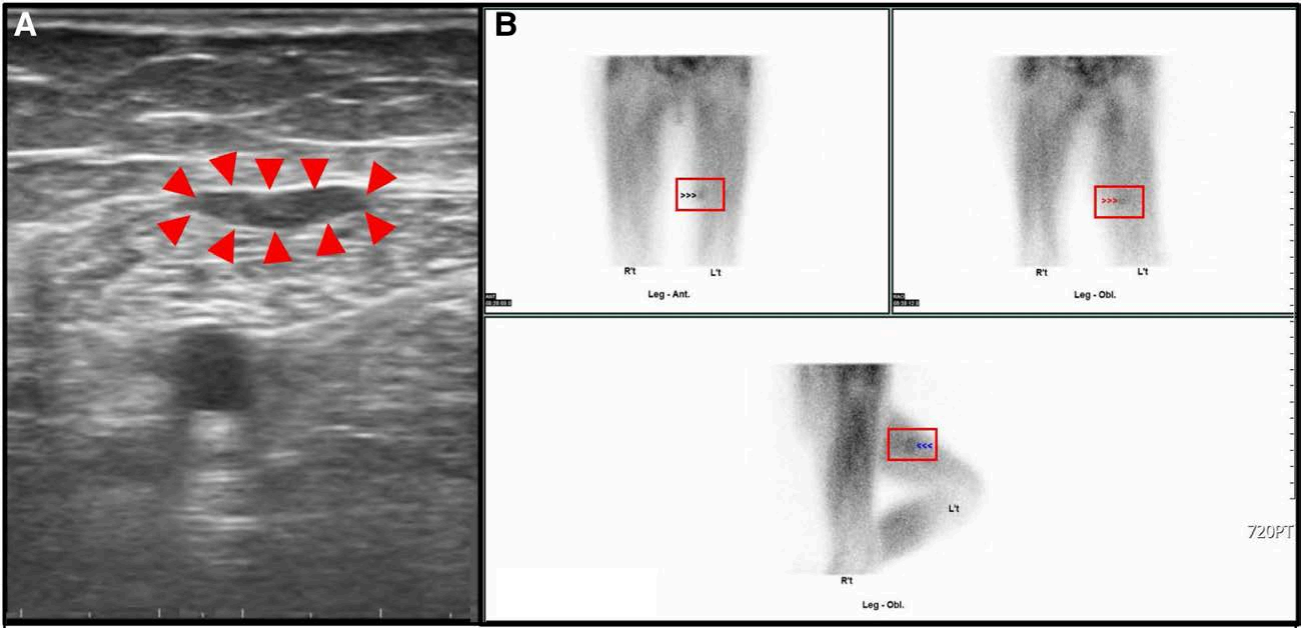
A. 2 hypoechoic lesions (1.75 cm × 0.57 cm, arrow head) respectively were noted on the left medial thigh in intraoperative sonography before second operation. Figure 3B. The dual phase Tc-99m MIBI parathyroid scintigraphy showed abnormal uptake on left thigh before second operation. (square frame).

**Figure 4.**
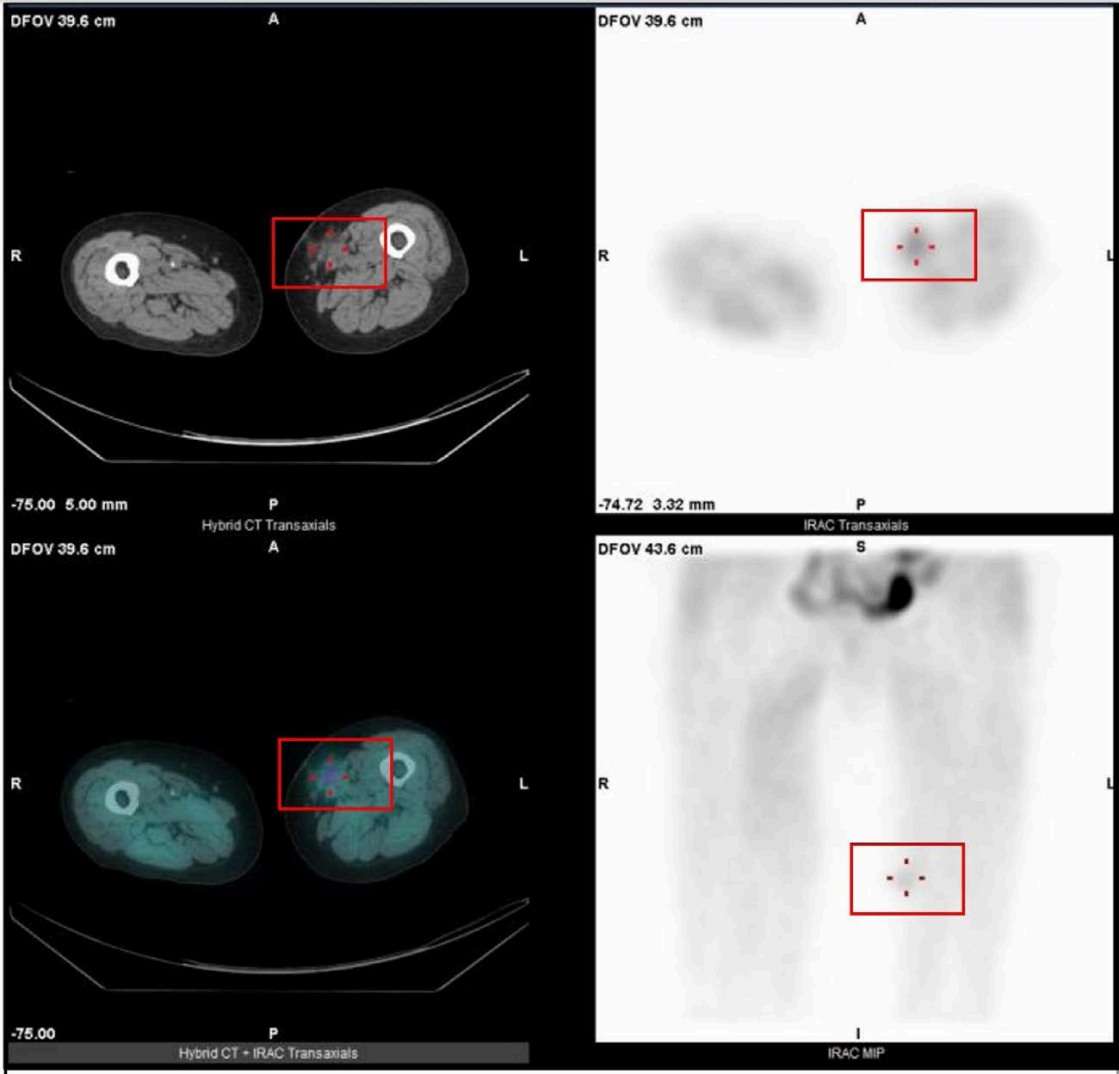
SPECT showed accurate position abnormal uptake at subcutaneous layer at lower medial aspect of left thigh. (square frame).

**Figure 5.**
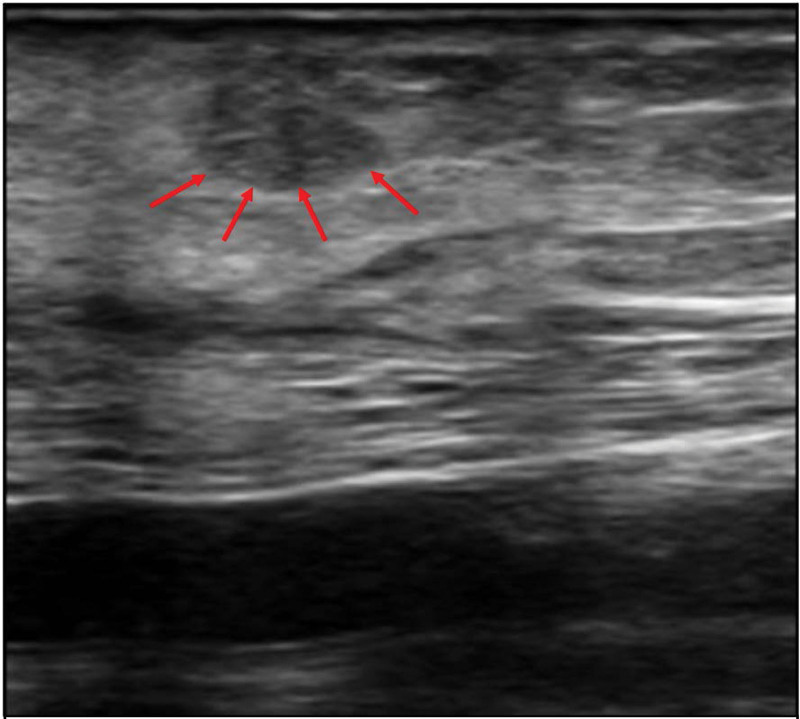
Intraoperative ultrasonography at secondary operation showed a hypoechoic lesion (2 cm × 1.4 cm × 0.6 cm, arrow), which was 3 times of initial size. (figure 1. Arrow).

**Figure 6.**
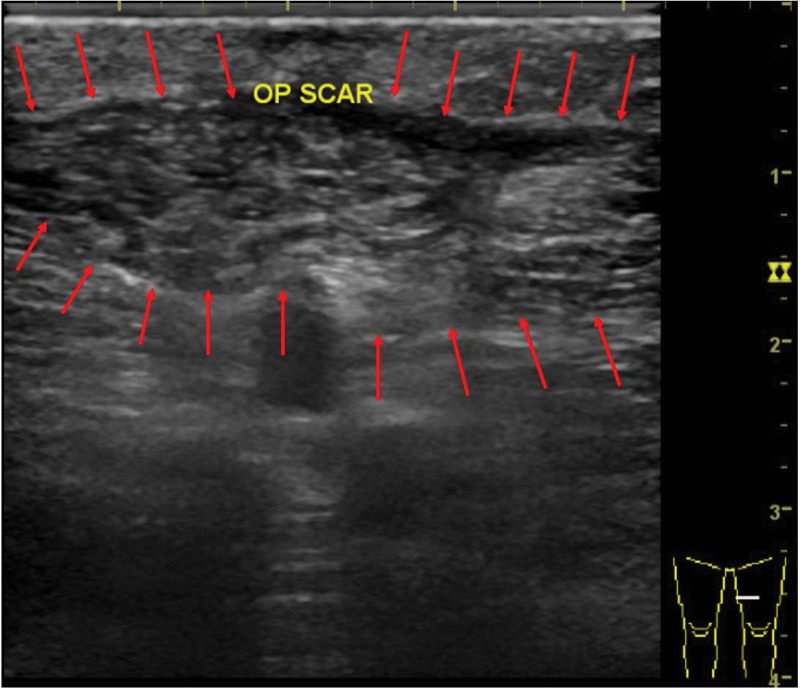
After follow-up 10 month, there was only scar at the operation site. No other residual parathyroid tissue seen in sonography.

The patient received an excision of transplant parathyroid tissue on left thigh on 20210812. The sonography before the surgery showed 2 masses (see Fig. 1, which showed 2 masses on the left medial thigh.). However, the small 1 of the 2 mass was too small to find in the operation after exploration. It was not definitely identified under sonography during operation and was left behind with the idea that even if the small remnant tissue not being removed might not be harmful afterwards. (see Fig. 2, which showed the larger parathyroid mass during first operation.) However, after surgery, laboratory investigation showed iPTH 1308.4 pg/mL on the next day. At the follow-up outpatient department, the value of iPTH was 806.53 pg/mL in the following week. However, the value elevated gradually after a week. To check if there was remnant secondary hyperparathyroid tissue, ultrasonography was performed and 2 hypoechoic lesions, which were 1.75 cm × 0.57 cm was noted on the left mild thigh adjacent but more medial and posterior to previous operative site (see Fig. 3A, which showed 2 hypoechoic lesions on the left medial thigh adjacent to previous operative site in ultrasonography.). Consideration of remnant parathyroid tissue, the dual phase 99m Tc-methoxy isobutyl isonitrile (Tc-99m MIBI) parathyroid scintigraphy was performed. The scintigraphy showed no parathyroid adenoma in the neck or chest. However, there was abnormal uptake on the left thigh in the dual phase Tc-99m MIBI parathyroid scintigraphy. (see Fig. 3B, which showed abnormal uptake on the left thigh in the dual phase Tc-99m MIBI parathyroid scintigraphy.) The single positron emission computed tomography (SPECT) showed suspecting parathyroid adenoma in the subcutaneous layer. (see Fig. 4, which showed suspecting parathyroid adenoma in the subcutaneous layer in the SPECT.) (see supplement 2, Supplemental Digital Content, http://links.lww.com/MD/I222, which presented patient’s symptom and persistent hyperparathyroidism after first surgery) Surgery for 2 lesions resection was arranged on 20210916. Intraoperative ultrasonography showed a hypoechoic lesion, which was 3 times of initial size. (see Fig. 5, which showed a hypoechoic lesion in intraoperative ultrasonography in second operation). The value of iPTH was surveyed immediately and dropped by two-third followed by gradual reduction in the following weeks. The calcemia and phosphatemia were back to normal gradually. There was scar on the operation site after ten months. (see Fig. 6, which showed scar on the operation site after 10 months.) During the follow-up AT outpatient department for 10 months until now, the value of iPTH dropped persistently within normal range and normal calcium level with regular daily supplement. (see supplement 3, Supplemental Digital Content, http://links.lww.com/MD/I223, which presented patient’s response after secondary operation and follow-up for more than 1 year.)

## 3. Discussion

Due to chronic kidney disease, the function of calcium reabsorption and phosphate excretion was decreased. Therefore, secondary hyperparathyroidism is one of the complications of chronic kidney disease. According to KDIGO 2017 clinical practice guideline update for the Diagnosis, evaluation, prevention, and treatment of CKD-MBD, when patients with CKD G3a–G5D with severe hyperparathyroidism who fail to respond to medical or pharmacological therapy, they suggest parathyroidectomy (2B).^[1–3]^

In Taiwan, parathyroidectomy with autotransplantation (AT) was suggested for most secondary hyperparathyroidism patients. Forearm AT after parathyroidectomy has turned into the standard method for secondary hyperparathyroidism treatment in chronic kidney disease patients. Surgical success of secondary hyperparathyroidism was associated with an intraoperative predictive criterion of iPTH 10 minutes ≤ 314.5 pg/mL or relative-iPTH 10 minutes ≤ 12.4%.^[4]^ There are 3 transplant methods including muscle, subcutaneous, and muscle plus subcutaneous. The efficiency differs from methods. According to the efficacy evaluation of the operation including preoperative and postoperative biological characteristics such as PTH, serum calcium, and serum phosphorus, the most efficient method was muscle plus subcutaneous, which also has beneficial on clinical outcomes and quality of life.^[5]^ However, recurrence of secondary hyperparathyroidism in patients after total parathyroidectomy with AT represents a major diagnostic and therapeutic challenge.^[6]^ Type 2 parathyromatosis would be caused by either the spillage or seeding of the parathyroid tissue during TPTX with AT.^[7]^ The recurrence rate was about 14.2% (16/112). All of these 16 patients received reoperation for forearm-autotransplant hyperparathyroidism. The histologic result of the autotransplants showed hyperplasia or an adenoma of the parathyroid gland particle. Although the recurrence rate was not extremely high, the reoperation would elevate risk including possible morbidity.^[6,8]^ According to a systematic review and meta-analysis, compared with TPTX + AT, patients in the TPTX group had lower rates of recurrence. No significant difference was found for “symptomatic improvement,” “complications,” “drug requirements,” and “hospital stay” (*P* > .05).^[6]^ Recurrence rates of 10–80% was reported in TPTX + AT (usually on the forearm).^[2]^ These findings indicate that TPTX is superior to TPTX + AT and demonstrate that TPTX + AT should be abandoned as a treatment of renal hyperparathyroidism for frequent recurrence.^[9]^

However, there was no guidelines on the management of recurrent hyperparathyroidism. But autograftectomy was recommended for patient with PTH > 800 pg/mL and hypercalcemia and/or hyperphosphatemia unresponsive to drug therapy.^[3]^ A single-surgeon retrospective cohort study showed incidence of autograftectomy was greater in the intramuscular autotransplant group (15/65) than the subcutaneous group (14/823). Besides, the incidence of repeated autograftectomy was 4 of 65 in the Intramuscular group and 1 of 823 in the Subcutaneous group, which means the cumulative frequency of the autograftectomy was greater in the intramuscular autotransplant (11.6 vs 3.1% at 6 years, *P* < .001). Their conclusion presumed that the recurrent rate was greater in the muscular autotransplant group.^[10]^ Lowering the recurrence rate of TPTX + AT was important for both surgeons and patients. Some techniques could assist us to achieve the goal. For example, we can use ultrasonography or computed tomography image at parathyroid region and graft area to detect any residual or hyperplasia of graft respectively before reexploration.^[2,3,7]^ But such studies cannot detect the atopic parathyroid tissue nor careless droppage of small pieces of parathyroid tissue when performing AT. On the other hand, 99mTc-sestamibi dual-phase imaging with SPECT/CT to reveal any intense radioactivity. Compared with planar imaging and SPECT alone, Tc-99m MIBI can yield higher accuracy of localization and fewer false-positive findings for parathyroid adenomas.^[7,11]^ Take our case for example. Recurrent hyperparathyroidism happened after 3 months post parathyroidectomy. As no abnormal lesion at neck region and nodular enlargement of graft tissue by ultrasonography, hyperplasia of transplanted parathyroid tissue was attributed to be the cause of recurrent hyperparathyroidism. Although autograftectomy revealed good response initially. The problem returned when the separate disseminated parathyroid tissue adjacent to the main graft became hyperplastic.

Due to underestimation, the recurrent site by taking only physical examination and sonography to localize the transplantation followed by inadequate removal of recurrent transplant tissue. After 1 week post the removal surgery in our case, hyperparathyroidism happened again. At this time, to find remnant parathyroid tissue, dual phase Tc-99m MIBI parathyroid scintigraphy was performed and intensive radioactivity around the autografted site was identified. Therefore, reoperation for removal of autografted tissue was done with Tc-99m MIBI parathyroid scintigraphy. After the surgery, PTH dropped dramatically, and showed normal value in 2 weeks after the surgery, which means success of removal of autografted tissue.^[2]^ If we used Tc-99m MIBI parathyroid scintigraphy before the first removal of autografted surgery, we could have removed autograted tissue as clearly as we could to prevent secondary recurrence. Tc-99m MIBI may also be useful to detect the other atopic parathyroid tissue in retro-sternum or other unrelated areas. Besides the assistance of image, the surgeons should be meticulous when performing the operation. When performing AT, contamination should be avoided during the operation. On the other hand, if the autograftectomy is needed, wide excision should be considered in order to prevent the inadequate evacuation of all parathyroid tissue and left remnant parathyroid gland tissue around the transplant site as in our case.

## 4. Conclusion

In conclusion, we strongly suggest to use Tc-99m MIBI for preoperative preparation for better identification of a disease site and SPECT/CT will give us more accurate localization of the residual parathyroid tissue. Tissue handling meticulously to prevent droppage of small smattered tissue rather than transplanted area may help detection and total excision of grafted tissue when needed for surgical intervention. Regarding the operative procedure for secondary hyperparathyroidism, the continuation of traditional TPTX + AT should be discussed more detail and further large randomized controlled trial study should be undertaken.

## Author contributions

**Conceptualization:** Wen Yao Yin.

**Formal analysis:** Wen Yao Yin.

**Investigation:** Wen Yao Yin.

**Project administration:** Wen Yao Yin.

**Resources:** Wen Yao Yin.

**Supervision:** Wen Yao Yin.

**Visualization:** Xin-Ling Guo.

**Writing—original draft:** Xin-Ling Guo.

**Writing—review and editing:** Xin-Ling Guo, Wen Yao Yin.

## Supplementary Material
