# Supplementary figures and images for: A preoperative parathyroid scan is important for the total removal of the transplanted parathyroid tissue in recurrent secondary hyperthyroidism: A case report and literature review
Source: Medicine (Baltimore). 2022 Dec 23;101(51):e32453. doi: 10.1097/MD.0000000000032453 (PMC9794238; doi:10.1097/MD.0000000000032453)

## Slide 1
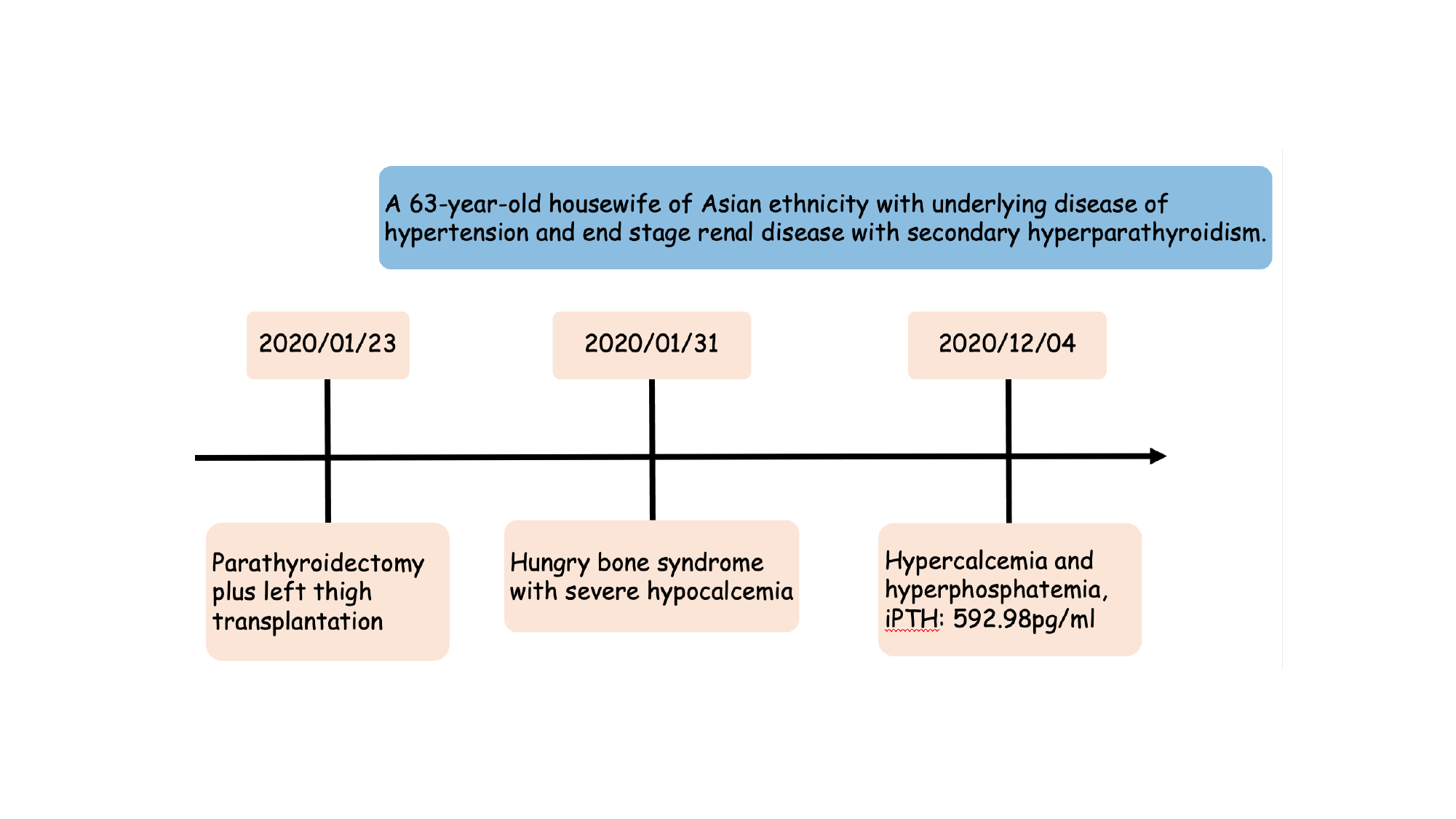

Supplement: Supplementary file 1 [file medi-101-e32453-s001.pptx]

## Slide 1
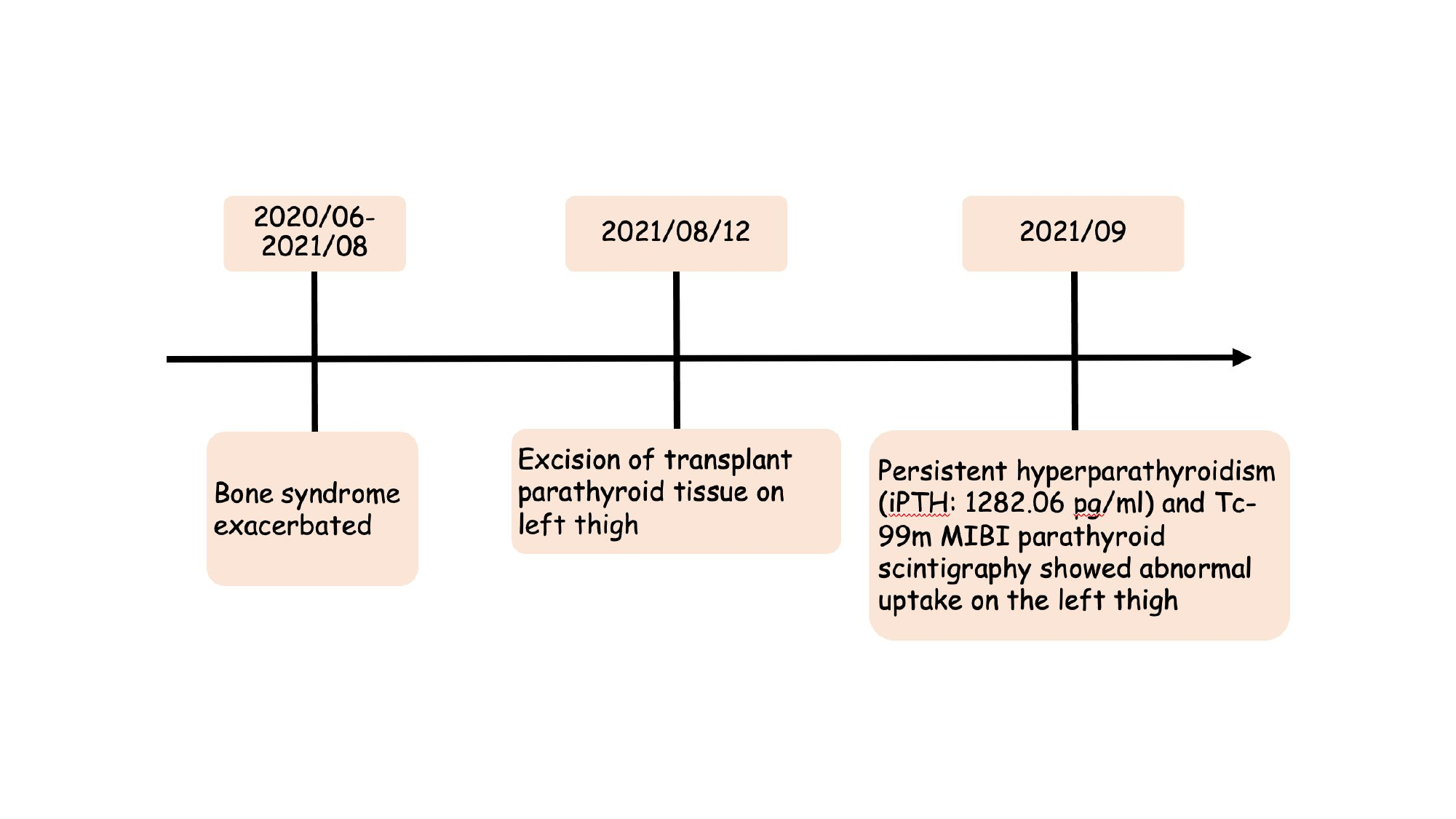

Supplement: Supplementary file 2 [file medi-101-e32453-s002.pptx]

## Slide 1
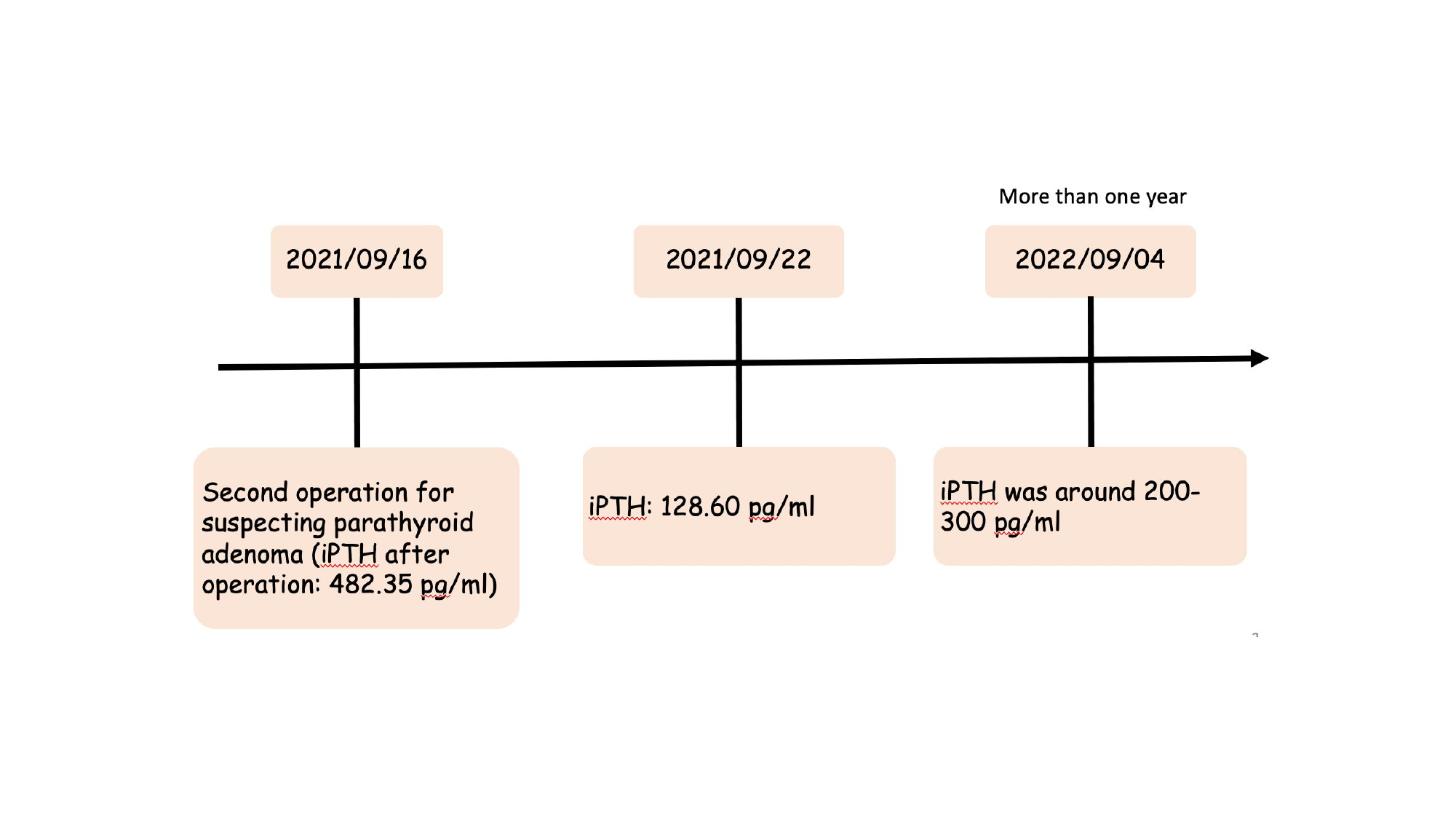

Supplement: Supplementary file 3 [file medi-101-e32453-s003.pptx]
